# ‘*Scars: How Our Wounds Make Us Who We Are’*: Improving appearance-based stigma, conceptualisation of beauty and body esteem through a documentary

**DOI:** 10.1177/20595131231205398

**Published:** 2023-11-16

**Authors:** Abigail Mathews, Bruna Costa, Aida Mikkola, Diana Harcourt

**Affiliations:** The Centre for Appearance Research, 1981 University of the West of England, Bristol, UK

**Keywords:** Visible difference, scars, stigma, appearance, intervention, media

## Abstract

**Introduction:**

Those with an altered appearance as a result of injury, health condition or treatment can face stigma, which may contribute to adverse psychosocial outcomes. However, current research tends to focus on supporting individuals themselves to cope, rather than targeting societal stigma. This study aimed to reduce societal stigma towards this group, through the use of a documentary about people with scars.

**Methods:**

146 adults completed questionnaires before and after viewing the documentary.

**Results:**

After viewing, participants had reduced self-reported intentions to behave in a stigmatising way towards those with visible differences, broader conceptualisation of beauty, and more positive body-esteem. Qualitative data also suggested further positive impacts.

**Conclusion:**

Those with visible differences (for example scars) are subject to societal stigmatisation which perpetuates psychological and social problems. Therefore, alleviating social stigma through the media, as demonstrated through the documentary in this study, may improve the lives of those living with visible differences.

**Lay Summary:**

People with an altered appearance or scars as a result of injury, health condition or treatment can face stigma, which may contribute to harmful psychological and social outcomes. However, current research tends to focus on supporting affected individuals themselves to cope, rather than targeting societal stigma. This study aimed to find out whether a documentary about people with scars was successful at reducing viewers’ stigma towards this group. A group of 146 adults completed questionnaires before and after viewing the documentary. After viewing, questionnaires indicated that participants had reduced intentions to behave in a stigmatising way towards those with visible differences. Furthermore, they also viewed a broader range of appearances as beautiful and felt more positive about their own bodies. Comments and feedback from participants also suggested further positive impacts. Those with visible differences (for example scars) are subject to societal stigmatisation which can cause and worsen mental health problems. Therefore, alleviating social stigma through the media, as demonstrated through the documentary in this study, may improve the lives of those living with visible differences.

## Introduction

The term ‘visible difference’ refers to an appearance that is notably different to the ‘norm’, typically as a result of injury or congenital or acquired health conditions, and includes scarring of any sort.^1^ As ‘visible difference’ can be difficult to define, estimates of the number of individuals living with a visible difference range between 2% and 20% of the UK population.^2,3^ Having a visible difference can have an adverse psychosocial impact for some affected, including depression, poor body image, low self-esteem, social isolation and a poorer quality of life.^1,4–6^ This may be because those who look ‘different’ can experience negative social encounters, including unwanted questions, staring, unkind comments, discrimination in different contexts (including in school and the workplace), and general societal stigmatisation.^7,8^

One explanation for this stigma is intergroup anxiety,^9^ whereby those with visible differences are seen as part of an ‘outgroup’ and are consequently avoided by those without. This may be due to evolutionary explanations, for example disease avoidance, whereby avoidance is activated through visibly perceiving signs that connote disease, though these are often inaccurate, especially in modern society.^10^ Another cause of this anxiety may be the learning of negative stereotypes, which are perpetuated through media depictions of those with differences.^11–13^

Intergroup anxiety can be overcome through many components, two of which are knowledge about the outgroup and contact with those in it.^9^ Therefore, providing education and a means of contact with the outgroup allows for individuals to readjust negative assumptions about those with differences, potentially reducing stigma against them. Despite this, most interventions in the field of visible difference centre around supporting the individuals themselves to better cope with challenges,^6,14^ rather than challenging and changing the societal attitudes that cause stigma.

## The role of the media

The media is a key source of appearance-based messaging, and thus a prominent way in which appearance-based norms and ideals are communicated.^15^ Appearance-based norms and ideals refer to societal pressures that govern the way individuals feel they should look. For example, in the UK, women experience pressure to be slim, whilst the ‘desired’ for men is to be lean and muscular.^16^ Television, movies, magazines, and social media are all significant in relation to communicating appearance-based norms and ideals in the general population.^17^ Exposure to media depictions of appearance norms and ideals may contribute towards appearance dissatisfaction.^18,19^ Problematically, this has been associated with adverse health and psychosocial outcomes including disordered eating, smoking, and drinking, decreased physical activity,^20,21^ poorer academic performance,^22^ and depression and low self-esteem.^23^

In addition to the appearance-related pressures facing the general population, those who have a visible difference often face additional challenges which can be exacerbated by the media. People with visible differences are rarely represented in the media,^11^ meaning there is limited visibility and normalisation of difference. Furthermore, existing media portrayals of visible difference often reinforce incorrect assumptions and negative attitudes.^12^ For example, visible differences such as scarring are often used in film and television as a reason for a character to be bitter, evil or a victim.^12,13^

There is a need for easily and widely accessible interventions aiming to promote understanding and acceptance of appearance diversity. Such interventions may work to reduce stigma and thus improve the experience of those living with, and affected by, visible differences. The media may be a particularly powerful avenue for these, due to its ability to increase the visibility of differences and thus work to normalise them.^12^

## The efficacy of media interventions at promoting acceptance of appearance diversity

To date, various media campaigns, including on TV and social media, have aimed to promote the acceptance of appearance diversity including visible differences.^13^ However, until now, the impact of these campaigns on people's attitudes towards their own and others’ appearance has rarely been evaluated. Although this recently emerging area of research is currently limited, available results are promising. Social media messages combining educational elements in conjunction with positive imagery of people with an appearance-altering condition (vitiligo) have been shown to be effective at reducing unaffected participants’ stigma towards differences.^24^ Stone and Fisher used indirect contact through audio and video personal narrative clips of people with facial differences and found participants had more positive assumptions of the target individuals’ personal and social skills after viewing either type of content.^25^

## The efficacy of media interventions at promoting appearance-related satisfaction

In addition to reducing social stigma and improving the social experiences of people with visible differences, media interventions aimed at promoting acceptance of appearance diversity may also have a beneficial impact on how members of the general public who do not have visible differences/an altered appearance feel about their own looks. Recently, media such as film and games including more diverse appearances in terms of weight and body shape, have shown efficacy at improving body satisfaction (how an individual feels towards their body's appearance), appearance-ideal internalisation (the extent to which an individual subscribes to socially defined ideals of appearance), and negative affect (adverse feelings).^26,27^ Further, global advertising campaigns are increasingly including people with a greater range of appearance diversity, with recent research suggesting they can be effective in improving self-esteem and positive affect in the general population.^28^

## Aims

This paper reports a study that aimed to evaluate the impact that watching a documentary about people with scars had on viewers’ intentions to behave in a stigmatising way towards those who look different, and their evaluations of their own appearance. In addition, the paper also aimed to consider whether low-level interventions such as this are a viable option for promoting acceptance of appearance diversity in society.

## Hypotheses

*H1. After viewing the documentary, participants’ intentions to act in a stigmatising way towards those who look different will decrease.*
*H2. After watching the documentary, participants will have a broader conceptualisation of beauty.*
*H3. After watching the documentary, participants’ body esteem will increase.*

## Method

### The documentary

*‘Scars: How Our Wounds Make Us Who We Are’* (referred to from hereon as ‘Scars’) is a 20-min documentary-style film which follows the personal stories of five adults with scars. It showcases each individual's story about their scarring, exploring their lived experience through personal narratives. The documentary was published in April 2020 and is freely available online [https://www.youtube.com/watch?v=d03vGv5K8qw&t=2s]. The research team had no input in creating the documentary.

Further information about the adults seen in ‘Scars’ can be found in Table 1.

**Table 1.** Descriptions of individuals featured in the ‘Scars’ documentary.

Name	Gender	Position of scar on body	Cause of scar
Pete	Male	Hand	Congenital condition
Christina	Female	Upper arm, covered by tattoo.	Domestic abuse/violence
Helen	Female	Forearms	Self-harm
Cordelia	Female	Scalp	Brain tumour surgery
Simon	Male	Across face and one eye	Military conflict

Within the documentary, the individuals reflect upon their experiences with others, such as positive reactions from friends and family, the impact of their scars in their personal relationships, and the reactions, impressions and assumptions from other people. Furthermore, their stories relate to overcoming adversity and include messages of strength and bravery, for example that the scar is symbolic of winning a battle. Finally, the individuals each signify their scars are now a part of who they are and tell true stories about a person.

Woven into their stories, contributors tell the viewer how their scars were acquired and the impact they have had on their lives, using close-up imagery of the scars themselves. Furthermore, the documentary works to humanise the contributors and their scars by providing opportunities for the viewer to connect and relate on a personal level, for example through discussing familial and romantic relationships, friendships, hobbies, employment, confidence, and self-esteem. The discussion of these topics is coupled with everyday life shots, for example of an individual gardening or spending time with their children. Further, the five individuals are varied in terms of ethnicity, gender, age-range, and type of scarring, which arguably offers more opportunities for a range of audiences to relate.

### Design

This study was carried out with two cohorts of adults: a general population sample (cohort 1) and a larger student sample (cohort 2).

Institutional ethical approval was obtained from the Faculty Research Ethics Committee at the University of the West of England. A pre-/post- design was selected to assess the impact of watching the documentary. Online surveys were designed to capture outcomes. The pre-documentary surveys included demographic information and standardised outcome measures (detailed below), before directing participants to watch the full documentary. Immediately after watching ‘Scars’, respondents were asked to complete the standardised outcome measures again, as well as provide additional feedback specifically related to the film. This study was run with two cohorts. In the first cohort, participants watched an online livestream of the documentary. In the second, participants were directed to watch the film online.

In addition to the survey, online data related to the documentary (including comments and other web analytics) were also collated and analysed in order to assess the broader public's reactions to ‘Scars’.

### Survey outcome measures

Outcome measures were chosen to assess respondents’ intention to engage in stigmatising behaviour towards those with a visible difference, as well as the ways respondents evaluated their own appearance and appearance more generally (including other people's appearance). The following well-established standardised measures were used:

*Stigmatising behaviour.* An adapted version of the Perceived Stigmatisation Questionnaire (PSQ)^29^ was used to evaluate respondents’ intention to act in a stigmatising way towards those with visible differences. Participants were asked to respond in relation to someone with a visible difference or scar, for example “I would avoid looking at them”. Items are rated on a 5-point Likert scale (1 = never, 5 = always), and a higher score indicates higher levels of intention to engage in stigmatising behaviours.

*Conceptualisation of beauty.* Seven items from the 9-item version of the Broad Conceptualisation of Beauty Scale (BCBS)^30^ were used to assess how respondents conceptualise beauty. Questions were adapted to not focus solely on women and body shape/size. Items such as “Even if a physical feature is not considered attractive by others or by society, I think that it can be beautiful” are rated on 7-point Likert scale (1 = strongly disagree, 7 = strongly agree). Higher scores indicate a greater ability to broadly conceptualise beauty.

*Body esteem.* The appearance subscale of the Body Esteem Scale for Adolescents and Adults (BES-AA)^31^ was used to evaluate respondents’ subjective satisfaction with their own appearance. It consists of 10 items, such as “I worry about the way I look”. Items are rated on a 5-point Likert scale (0 = never, 4 = always). Negative items are reverse scored, and a higher score indicates higher body esteem.

### Online data

Data was extracted from publicly available platforms including YouTube, Vimeo, Twitter, Instagram, Facebook, The Guardian newspaper's webpages, and Reddit. A search using the title of the documentary (*Scars: How Our Wounds Make Us Who We Are*) was conducted in August 2020, four months after the documentary's release in April 2020. Data extracted included the number of times the documentary was watched, liked and shared, and social media comments were collated. A content analysis was conducted on the comments in order to evaluate how the documentary was received by the public beyond the participants within this study.

### Recruitment

*Cohort 1 (C1).* The study was first advertised to the general public, predominantly through relevant social media and other online platforms (e.g., the University of the West of England and research centre, and UK charitable organisations that work to support people affected by visible difference) from April 2020 to July 2020.

*Cohort 2 (C2).* The study was then advertised to a student sample, on the University of the West of England Undergraduate Psychology Student Participant Pool in February 2021. Students were given course credits for taking part.

### Participants

*C1.* A total of 43 people from the general population responded to the online survey. Most were female (75.4%, *n = *32), 10 (23.3%) were male and one respondent did not report their gender (2.3%). Participants had a mean age of 38.72 (SD = 14.49), ranging from 17–70 years. Most respondents (86.0%, *n = *37) indicated that they, or someone they knew had a visible difference or scar. Of these, 36 reported having a scar themselves. Further information can be found in Table 2.

**Table 2.** Causes of scarring amongst participants.

	**C1 *n* (%)**	**C2 *n* (%)**
**Participants who reported having a visible difference or scar**	36 (83.72%)	56 (54.36%)
**Reason for scarring**		
Medical treatment or surgery	9 (25%)	8 (14.29%)
Injury, including stabbing, dog attack, burns or accident	4 (11.1%)	11 (19.64)
Self-harm	4 (11.1%)	3 (5.36%)
Congenital, including cleft lip and/or palate	3 (8.3%)	3 (5.36%)
Circumcision	1 (2.8%)	0 (0%)
Eczema	0 (0%)	1 (1.79%)
Multiple causes	5 (11.1%)	8 (14.29%)
Did not disclose	10 (27.8%)	22 (39.29)

*C2.* A total of 103 undergraduate students responded to the online survey, 80 female (77.7%); 23 male (22.3%). Participants had a mean age of 22.68 (SD = 0.73), ranging from 16–60 years. When asked whether they, or someone they knew had a visible difference or a scar, most respondents (69.9%, n = 72) said ‘yes’. Of these, 56 reported having a scar themselves. Further information can be found in Table 2.

### Analyses

Paired samples t-tests compared sample mean scores on stigamatising behaviour (PSQ), conceptualisation of beauty (BCBS) and body esteem (BES-AA), at time 1 (pre-documentary) and time 2 (post-documentary), for both the general population cohort (C1) and student cohort (C2).

Qualitative data (including that extracted from the surveys and the comments extracted from online platforms) were analysed by the second and third authors, BC and AM, using inductive content analysis.^32^ The data were read multiple times and a preliminary set of frequently occurring themes (codes) were identified and then applied to the full data. All codes were compared between researchers until agreement was reached. Frequency counts and example quotes were selected to demonstrate each code.

## Results

### Outcome measures

*Stigmatising Behaviour.* In both cohorts there was a significant difference between PSQ scores at time 1 and time 2: C1 time 1 (M = 1.86, SD = 0.532) and time 2 (M = 1.73, SD = 0.650); t(42) = 2.331, *P* = 0.025; C2 time 1 (M = 1.71, SD = 0.466) and time 2 (M = 1.50, SD = 0.415); t(102) = 7.515, *P* ≤ 0.001. Therefore, in both C1 and C2, respondents’ intentions to act in a stigmatising way towards those with visible differences reduced after watching the Scars documentary, supporting H1.

*Conceptualisation of Beauty.* BCBS scores at time 1 (M = 5.83, SD, 1.103) and time 2 (M = 6.01, SD = 1.134) were found to be significantly different in C1; t(42) = −2.344, *P* = 0.024. Similarly, in C2, BCBS scores at time 1 (M = 6.15, SD, 0.780) were significantly different to those at time 2 (M = 6.40, SD = 0.693); t(102) = −5.006, *P *≤ 0.001. In support of H2, in both C1 and C2 respondents’ ability to more broadly conceptualise beauty increased after watching the Scars documentary.

*Body Esteem.* In C1, participants’ body esteem scores (BES-AA) at time 1 (pre-documentary) (M = 2.08, SD = 0.926) were significantly different to those at time 2 (post) (M = 2.29, SD = 0.810); t(42) = −3.791, *P* ≤ 0.001. In C2, there was also a significant difference between the BES-AA scores at time 1 (M = 1.81, SD = 0.782) and time 2 (M = 2.39, SD = 0.504); t(102) = −13.078, *P* ≤ 0.001. In both C1 and C2, respondents’ body esteem scores were more favourable after watching the ‘Scars’ documentary, supporting H3.

### Other data

When asked whether they felt that watching the documentary changed their beliefs about or attitudes and/or reactions to scars, 36.3% (*n *= 15) of respondents in C1 and 55.3% (*n *= 57) of respondents in C2 said ‘yes’.

In both C1 and C2, at time 2 (post-documentary), respondents were asked further questions about the documentary. Table 3 provides a full overview of codes and exemplar quotes. Respondents who indicated that the documentary changed their beliefs, attitudes and/or reactions to scars reported various positive impacts of watching the documentary, which included increased acceptance of self and others, and positive comments about those featured in the film. Those who indicated that it did not change their beliefs, attitudes and/or reactions to scars cited already having positive views prior to seeing the film.

**Table 3.** Survey comments and themes.

**Theme**	**Exemplar Quotes**	**C1 N (%)**	**C2 N (%)**
**Question: Do you feel that watching the documentary changed your beliefs, reactions and/or attitude(s) to scars?**			
		Total: 41	Total: 100
Positive impact – more accepting of self	“More accepting of myself” “Kind of, slightly it definitely moved me towards the more positive / acceptance side”	3 (7.32%)	6 (6%)
Positive impact – hopes to achieve greater confidence/acceptance	“I really appreciated everyone's level of self-confidence… hope that one day I can be like that with my appearance, as well.” “It made me question my acceptance of my own scars and how I could reframe my experience”	3 (7.32%)	1 (1%)
Positive impact – thinking about scars differently	“I realise that having scars does not prevent your job prospects” “It made me think about the story behind the scar”	2 (4.88%)	34 (34%)
Positive comments about people in the documentary	“I was encouraged by the people in film and how they were still optimistic and confident…”	2 (4.88%)	4 (4%)
No impact/change – existing knowledge and attitudes/beliefs	“I was already pretty familiar with similar stories so watching the film didn't change much.” “it confirmed them” “No - I think I already had quite ‘positive'/holistic beliefs about scars.”	14 (34.15%)	26 (26%)
**Question: Do you feel that art mediums, such as films, are a good way to increase awareness and acceptance of appearance diversity?**			
		Total: 40	Total: 102
Importance of representation	“The more often you see diversity in the media, the more ‘normal’ and accepted it becomes and the easier it is to not see it as different in a negative light.”	5 (12.5%)	25 (24.5%)
Power of the media (using it for good)	“It normalizes our differences in context”	2 (5%)	21 (20.59%)
Media can educate	“Yes, because many people have no experience of these things and it’s a way of spreading knowledge and understanding” “They improve understanding and acceptance” “Yes I feel we could learn more using this method”	9 (22.5%)	7 (6.86%)
Benefits of art	“Art connects and heals us as human beings.” “People are more likely to think about them and be wiling to watch them.” “Good way of getting someone's emotions across”	6 (15%)	13 (12.75%)
Other art mediums	“…I don't have the patience to sit through media such as films/ videos unless I have to. I think other art forms would be a better viewing medium for me.”	2 (5%)	0 (0%)
Benefit of stories	“Yes. I like to hear voices and see images of experience. For me, having the person tell their story helps me to connect with them and understand more about them.” “Seeing people with diverse appearance talking about their experience helps people to relate and empathise”	7 (17.5%)	14 (13.73%)

Note: Answers extracted from a free text-box response, ‘Please explain your answer’.

Most respondents (C1 n = 39; 90.7%; C2 n = 101; 98.1%) agreed that art mediums, such as films, are a good way to increase acceptance of appearance diversity. Cited reasons included the importance of media representation, the power of the media to normalise difference and educate, the ability for the media to be engaging and emotionally powerful, and the benefit of personal stories.

### Web analytics and online comments

In July 2020, the documentary had been viewed 62,436 times (YouTube and Vimeo) and generated a total of 53 posts through Instagram (*n* = 9), Facebook (*n *= 12), Twitter (*n *= 29), and Reddit (*n *= 3). These posts and views received 2566 likes and 248 shares. Twitter accounted for more than half of these posts (52.72%) and nearly half of the shares (49.20%). Most likes were found on YouTube (29.54%) and Instagram (26.04%). Within these 53 posts there were 211 comments (YouTube (32.72%), Instagram (25.88%), Twitter (22.66%), Facebook (18.43%), Vimeo (2.30%)).

The 211 comments were further divided into 258 individual quotes, and a total of 15 codes were created from them (see Table 4). These included compliments about the documentary, its filming and production, or the people in the documentary, expressions of gratitude for the documentary, sharing of personal life experiences, and suggesting that there is a need for similar documentaries.

**Table 4.** Online comments.

**Codes**	**Exemplar quotes**	***n* (%) of total quotes**
**General compliments of the documentary**	“Just watched it - it's really gorgeous” “Beautiful film”	88 (34.11%)
**Compliments of the filming, editing, production and/or direction**	“Brilliant video, beautifully filmed and edited.”	5 (1.94%)
**Positive comments about the people in the documentary**	“Beautiful people” “I admire their courage”	33 (12.79%)
**Thankful for the documentary**	“What an excellent film thank you for making it” “We need more of this. Thank you.”	21 (8.14%)
**Sharing own experiences of scars**	“I have multiple scars on my body and each one comes with its own story…I don’t hide them because they are part of who I am.”	20 (7.75%)
**Sharing own experience of life more generally**	“I can feel it . . I have gone through all of this. For people you are just a joke kinda pointless.”	9 (3.49%)
**Need for similar documentaries and further discussion on related topics**	“Could have done with more things like this as a child growing up with extensive scarring, and especially as a teenager”	5 (1.94%)
**General comments about scars**	“Scars tell stories about what we’ve lived and who we are – or were. Some scars are visible; some are not.” “Scars tell us a lot deeper than just skin deep. It's the history of us making the best version of a person we are now”	15 (5.81%)
**Documentary looks interesting / must watch it**	“This looks very interesting” “Very cool. Want to watch this!”	11 (4.26%)
**Negative comments about the documentary**	“How about you get over it instead of writing an article about it?”	4 (1.55%)

## Discussion

The findings from this study suggest that a 20-min documentary, depicting the experiences of real people with real scars, had a beneficial impact on how people feel about their own appearance (body esteem), appearance in general (conceptualisation of beauty) and their views towards people with scars. Participants described how watching the documentary had made them more accepting of themselves, given them hope for becoming more self-confident, and prompted them to think about scars differently. They were positive towards media, like film, as a good way to increase acceptance of appearance diversity for many reasons, such as demonstrating representation, normalising difference, providing education, showcasing personal stories of real people, and doing so in a way that is engaging and emotionally powerful. Furthermore, qualitative data extracted from various online platforms showed mostly favourable comments about the documentary and people in it, suggesting that the general public (beyond those taking part in the study) found it acceptable and positive.

Findings from the current study suggest that watching ‘Scars’ reduced intentions to engage in stigmatising behaviour towards those who look different and do not fit the societal beauty ‘norm’. However, a large proportion of participants in C1 and C2 (36.3% and 55.3% respectively) stated that the documentary did not change their attitudes or beliefs towards scars. Those who elaborated on their answer stated that this was because they already held positive attitudes. The significant improvement on the PSQ in both cohorts, even when many participants stated it did not change their views, may indicate that watching ‘Scars’ improved stigmatising attitudes even in those with previous unstigmatised perceptions. Alternatively, it may highlight differences between explicit and implicit stigma within these groups. Further, the outcome measures used in this study are more comprehensive than the questions asked in the feedback questionnaire, and so may be more sensitive to nuanced changes in attitudes and stigma.

This supports previous work demonstrating the ability of media-based interventions to reduce stigma towards those with visible differences.^24,25,33^ Given the possible social challenges that those with altered appearances may experience,^8^ and the resulting negative impact on wellbeing,^1^ our findings suggest that the development and promotion of destigmatising interventions (like ‘Scars’) is a worthwhile effort.

Further, ‘Scars’ also led to viewers feeling better about themselves and their own appearance, as demonstrated by more positive body esteem results and qualitative feedback of the documentary. Traditional media often depicts ‘idealised’ appearances which, for most people, are unrepresentative and unattainable, expectantly resulting in feelings of body dissatisfaction.^34,35^ Scholars have previously expressed the urgent need for interventions to improve body image and body confidence.^36^ The current findings echo existing literature which demonstrate that exposure to diverse appearances in the media can have a positive impact on consumers’ feelings towards their own appearance,^26,27^ possibly through minimising comparisons with others.^37^ Specifically, similarly to previous research,^38,39^ the current study found that exposure to diverse appearances (in the form of people with various types of scars), had a beneficial impact on body-esteem.

### Implications for practice

Despite the long-documented ability of the media to cause body dissatisfaction,^18,19^ and stigmatise those who look different,^12^ this emerging area of research demonstrates the media as a potential tool to foster positive outcomes for both attitudes towards others and individuals’ own bodies. It is therefore important to consider both what makes a media intervention effective, and how to produce positive outcomes rather than causing harm. For example, previous research has demonstrated that although a documentary effectively reduced weight bias in viewers,^40^ a reality television programme significantly increased it,^41^ highlighting the importance of the messaging and context in which it is presented. Previous research suggests that both positive messages, and messages of overcoming adversity, are effective in reducing stigma towards others with visible differences.^25^ The stories within ‘Scars’ are framed positively and demonstrate individuals overcoming adversity. Although there are negative messages within these stories (e.g., struggles with self-harm and domestic violence), the overarching message of the documentary remains largely positive, and each story ends on a positive note. In previous literature, it has been suggested that endings may leave the strongest impression due to a type of recency effect,^25^ which may explain the current findings.

It has been previously suggested that stigma occurs due to intergroup anxiety and that two methods of reducing intergroup anxiety are (1) providing contact with the outgroup and (2) providing education about the outgroup.^9^ Providing indirect contact, for example through ‘Scars’, is suggested to avoid physiological stress that may be associated with direct contact with a stigmatised group.^25,42^ ‘Scars’ provides first-person narratives, which evidence suggests may be particularly effective at reducing appearance-based prejudice and discrimination in general population interventions.^25,43^ Further, ‘Scars’ educates viewers on the acquisition, management of and adjustment to various types of scars. Consistent with intergroup anxiety theory, previous research suggests that providing education about the target condition is an important aspect of reducing stigma towards those affected by it.^24,33^

In summary, the ‘Scars’ documentary provides positive messaging, personal narratives, education and visual exposure of scars, all of which may have contributed to the favourable findings. However, our study could not pinpoint which individual or combined elements led to the positive effect of the documentary. As such, further research is needed to better understand which elements of media-based interventions aimed at increasing acceptance of appearance diversity are most important for efficacy.

Beyond the content of the documentary, it would also be beneficial to learn how to fine-tune technical aspects of such interventions to maximise effectiveness. For example, the length of the video could be significant. A documentary similar in length to ‘Scars’ was found to effectively reduce weight bias in viewers.^40^ However, Stone and Fisher found that much shorter exposure (2two-minute narratives via audio or video) significantly increased positive personality assumptions and feelings about possible future interactions with people with facial differences.^25^ Alternatively, it may be the case that shorter clips with repeated exposure may be more effective.^24^ Conversely, it should also be examined whether it is possible to overexpose individuals to this interventional content. Future research should aim to determine which elements make these interventions effective, the optimum exposure needed to generate positive change and conduct follow-ups to determine the longevity of positive effects.

### Limitations

It is possible that people who chose to take part, particularly in C1, were already interested in the topic of visible difference and scarring, meaning they may not be representative of the broader population. Indeed, in C1, most respondents reported having a visible difference or scar (83.72%). However, the documentary had a similarly positive effect in C2, where considerably less participants reported having a visible difference or scar (54.36%). Describing scarring as a visible difference in this study may have encouraged a higher proportion of participants to self-identify as having a visible difference than would ordinarily be the case, meaning that the percentage of those with visible differences in our sample is likely to be a liberal estimate. Further, in addition to participants having visible differences and/or scars themselves, it is arguable that individuals who sign up to research on a voluntary basis may differ in terms of personality characteristics, motivations and beliefs which resultingly generate more favourable responses within psychological research studies than may be seen in the general population.^44,45^ Nevertheless, the authors propose that the significant pre- and post- differences found in both samples is promising.

It is important to note that the current study relied on self-report, which could have raised issues of demand characteristics and social desirability.^46^ Participants may have understood the intention of the study (pre- and post- assessment with ‘Scars’ being the stimuli) and provided responses which they felt would be viewed favourably by others. In addition, the adapted version of the PSQ was used to evaluate respondents’ *intention* to act in a stigmatising way towards those with visible differences, and not their *actual behaviour*. Whilst this relationship is not definitive and there are questions about the intention-behaviour link, behavioural science generally shows a strong association,^47^ and outgroup attitudes regarding future contact have been previously found to relate to actual contact.^48^

Despite these limitations, these findings are still helpful and promising with regards to informing the development of media-based interventions to promote acceptance of appearance diversity amongst the general population. More research is needed to better understand what aspects of such interventions result in efficacy. Furthermore, future research should use follow-ups in order to assess the long-term impact of such interventions. Additionally, work with other audiences and in different contexts is warranted. For example, it would be helpful to examine whether ‘Scars’ would be effective with young people in a school setting, since research suggests media interventions may be less effective in this population.^49^

### Conclusions

While the media can have a negative impact on individuals’ appearance satisfaction and stigma towards those who look different, it can also be used as a means to normalise, educate, and destigmatise. The present study addresses an important gap in the literature and demonstrates the effectiveness of a low-level intervention aiming to promote understanding and acceptance of appearance diversity. Watching ‘Scars’ led to participants feeling more positive about their own looks, whilst also viewing those with visible differences more favourably. This in turn may contribute to a reduction in stigma (and stigmatising behaviour), and fewer social challenges for those living with, and affected by, visible differences.
